# Cerebrospinal fluid-based spatial statistics: towards quantitative analysis of cerebrospinal fluid pseudodiffusivity

**DOI:** 10.1186/s12987-024-00559-z

**Published:** 2024-07-18

**Authors:** Yutong Chen, Hui Hong, Arash Nazeri, Hugh S. Markus, Xiao Luo

**Affiliations:** 1 Department of Clinical Neuroscience, University of Cambridge, Cambridge, UK; 2 Department of Radiology, The Second Affiliated Hospital of Zhejiang University School of Medicine, Hangzhou, 310009 Zhejiang China; 3 Mallinckrodt Institute of Radiology, Washington University School of Medicine, St. Louis, MO USA

**Keywords:** Cerebrospinal fluid, Dementia, Pseudodiffusivity, Glymphatic system, Intravoxel incoherent motion

## Abstract

**Background:**

Cerebrospinal fluid (CSF) circulation is essential in removing metabolic wastes from the brain and is an integral component of the glymphatic system. Abnormal CSF circulation is implicated in neurodegenerative diseases. Low b-value magnetic resonance imaging quantifies the variance of CSF motion, or pseudodiffusivity. However, few studies have investigated the relationship between the spatial patterns of CSF pseudodiffusivity and cognition.

**Methods:**

We introduced a novel technique, CSF-based spatial statistics (CBSS), to automatically quantify CSF pseudodiffusivity in each sulcus, cistern and ventricle. Using cortical regions as landmarks, we segmented each CSF region. We retrospectively analyzed a cohort of 93 participants with varying degrees of cognitive impairment.

**Results:**

We identified two groups of CSF regions whose pseudodiffusivity profiles were correlated with each other: one group displaying higher pseudodiffusivity and near large arteries and the other group displaying lower pseudodiffusivity and away from the large arteries. The pseudodiffusivity in the third ventricle positively correlated with short-term memory (standardized slope of linear regression = 0.38, adjusted *p* < 0.001) and long-term memory (slope = 0.37, adjusted *p* = 0.005). Fine mapping along the ventricles revealed that the pseudodiffusivity in the region closest to the start of the third ventricle demonstrated the highest correlation with cognitive performance.

**Conclusions:**

CBSS enabled quantitative spatial analysis of CSF pseudodiffusivity and suggested the third ventricle pseudodiffusivity as a potential biomarker of cognitive impairment.

**Supplementary Information:**

The online version contains supplementary material available at 10.1186/s12987-024-00559-z.

## Introduction

Cerebrospinal fluid (CSF) plays a critical role in clearance of toxins and the maintenance of optimal brain function [1]. The exchange of substances between CSF and interstitial fluid (ISF), as well as CSF motion through the brain ventricles and subarachnoid space, contribute to the homeostasis of the central nervous system [1]. While numerous studies have used non-invasive imaging methods to investigate CSF-ISF exchange, also known as glial-lymphatic (glymphatic) system, in neurodegeneration diseases [2, 3], there is a paucity of research exploring the role of CSF motion in the ventricles and subarachnoid space.

An important technique for quantifying CSF motion is low b-value diffusion magnetic resonance imaging (low-b dMRI). Low-b dMRI is sensitive to a wide range of incoherent motion (0.1–1000 × 10^− 3^ mm^2^/s), i.e., pseudodiffusivity [4], and captures the variation of CSF diffusion pseudodiffusivity at a microscopic level [5].

However, quantitative assessment of CSF pseudodiffusivity is challenging owing to marked individual variation in the anatomy of the CSF-filled sulci, cisterns and ventricles, and the difficulty of registering low resolution low-b dMRI signals onto a standard template for voxel-wise analysis. One study manually selected regions of interest in each CSF-filled sulci and cisterns [6] but used visual rating of low-b dMRI signals instead of quantitative analysis. The lack of quantitative approach to analyze CSF signals prevents investigating the role of CSF pseudodiffusivity in neurodegenerative diseases.

To address this issue, we developed a novel technique: cerebrospinal fluid-based spatial statistics (CBSS) to quantify CSF motion from low-b dMRI by parcellating CSF regions according to their underlying gray matter regions. Using a cohort of 93 participants with varying degrees of cognitive impairment, we demonstrated how CBSS gauged the regional variations of CSF pseudodiffusivity among sulci, cisterns and ventricles. Next, we investigated how CSF pseudodiffusivity is connected to aging and cognition. To obtain further insight into the driving forces behind CSF circulation, we investigated the relationship between CSF pseudodiffusivity and cerebral blood flow, which is influenced by cardiac and arterial pulsation [7, 8] and neurovascular coupling [9] and may serve as an indirect measure of a combination of these factors. We hypothesize that a slower CSF pseudodiffusivity would be linked to aging, reduced cognition and reduced cerebral blood flow.

## Materials and methods

### Cohort selection

Patients were recruited from a memory clinic at the Second Affiliated Hospital of Zhejiang University School of Medicine. Inclusion criteria encompassed individuals with mild cognitive impairment (MCI) or Alzheimer’s disease (AD) dementia, which was ascertained by experienced neurologists. MCI was defined as the presence of demonstrated cognitive impairment as indicated by Wechsler Memory Scale Logical Memory (WMS-LM) delayed recall performance [10] and a Clinical Dementia Rating scale (CDR) score of 0.5 [11, 12], but with the preservation of daily living activities and the absence of dementia. AD dementia was diagnosed when patients met the following criteria: mini-mental state examination (MMSE) score [13] ≤ 26, CDR score ≥ 0.5, and met the NINCDS/ADRDA probable AD criteria [14]. Normal control participants were also recruited from the community or the relatives of patients. Inclusion criteria were (1) with a CDR score of 0; (2) MMSE score between 24 and 30 (inclusive).

Exclusion criteria for both patient and normal control groups were: (1) significant medical, neurological (with the exception of probable AD), or psychiatric conditions; (2) a documented history of substantial head trauma; (3) the use of non-AD-related medications known to influence cerebral function; (4) clinical depression defined by Geriatric Depression Scale [15] ≥ 5; (5) a history of alcohol or substance abuse; and (6) left-handedness.

Participants underwent one hour of brain MRI including T1-weighted MRI, T2 fluid attenuation inversion recovery (T2-FLAIR), diffusion MRI (dMRI), low-b dMRI and arterial spin labelling. They undertook one hour of cognitive assessments including MMSE, Montreal cognitive assessment (MOCA) [16], WMS-LM to assess short term memory (STM) and long term memory (LTM), and trail making test A (TMTA) [17, 18] to assess processing speed. In MMSE, MOCA, STM and LTM, higher scores indicate better memory. In TMTA, the longer a participant took to complete the test, the worse his/her processing speed was.

### Imaging parameters

MRI scans were conducted using a 3 Tesla General Electric Discovery MR750 scanner. T1-weighted imaging was acquired using 3D spoiled gradient echo sequences with the following imaging parameters: echo time (TE): 3 ms, repetition time (TR): 7 ms, voxel size 1.20 × 1.02 × 1.02 mm^3^, acquisition matrix size: 196 × 256 × 256. Axial 2D T2-FLAIR images: TE: 158 ms, TR: 11,000 ms, voxel size: 0.86 × 0.86 × 4.00 mm^3^, acquisition matrix size: 256 × 256, 42 slices. Single shell dMRI was acquired with a b-value of 1000 s/mm^2^, 30 gradient directions and under posterior-to-anterior encoding. Five volumes with a b-value of 0 s/mm^2^ were acquired. Each dMRI volume was acquired with the following parameters: TE 81 ms, TR 8000 ms, voxel size 2.0 × 2.0 × 2.0 mm^3^, acquisition matrix size: 128 × 128, 67 slices. Low-b dMRI was acquired with b-values of 0, 10, 20, 30, 50, 70, 90, 110, 130, 150, 170, 200, 500, 800, 1000 and 1500 s/mm^2^ with each b-value being acquired under 3 different gradient directions. The imaging parameters were: TE 75 ms, TR 3000 ms, voxel size 2.0 × 2.0 × 4.0 mm^3^, acquisition matrix size: 128 × 128 × 28. All dMRI scans were acquired under posterior-to-anterior phase encoding direction. Reverse phase encoding images were not acquired and distortion correction was not performed. Pseudo-continuous arterial spin labelling was acquired with: TE: 10 ms, TR: 4632 ms, voxel size 1.88 × 1.88 × 4.00 mm^3^, acquisition matrix size: 128 × 128, 36 slices, post-labelling delay: 1500 ms. All participants were scanned between 8-10am and were instructed to be awake during the scan.

### CBSS

#### Sulcus Altas

A sulcus-specific gray matter atlas was created in the MNI space (https://fsl.fmrib.ox.ac.uk/fsl/fslwiki/Atlases) by two radiologists, where the gray matter regions in this atlas were labeled in accordance to their overlying sulci and cisterns (Figure S1). Sixteen regions (8 sulci and 8 cisterns) were delineated [19]. Smaller sulci were not included due to individual variations and the limited resolution of low-b dMRI images. The “suprasellar” cistern in the atlas encompassed the regions of suprasellar, suprachiasmatic, Sylvian and interpeduncular cisterns. The superior longitudinal fissure was divided into the anterior and posterior parts by the pars marginalis of the cingulate sulcus.

#### Pseudo-T2 image

**Fig. 1:**
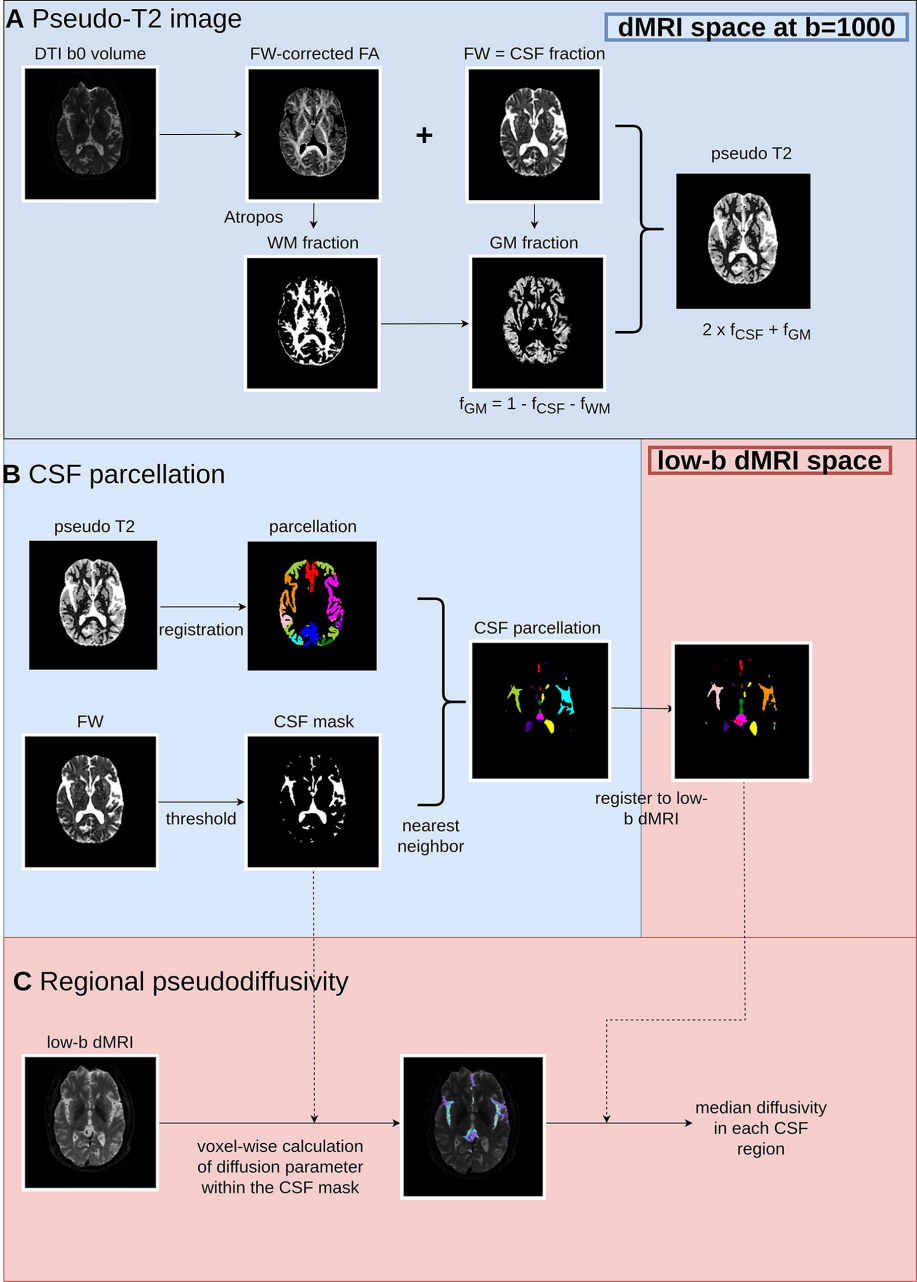
CBSS algorithm. (**A**) Pseudo-T2 image generation. (**B**) in the panel “parcellation” and “CSF parcellation”, each color represents a sulcus/cistern. Left and right structures are colored separately. (**C**) the color in the second panel represents the pseudodiffusivity in the CSF region with blue being higher pseudodiffusivity and purple being lower pseudodiffusivity. Abbreviations: GM: gray matter, CSF: cerebrospinal fluid, WM: white matter, FW: free water, FA: fractional anisotropy

Motion correction was performed on the dMRI images using the FSL software [20]. Maps of free water and free water corrected fractional anisotropy (cFA) were obtained from dMRI [21]. Skullstripping was performed on the free water map using FreeSurfer [22]. The free water map served as a proxy of the CSF portion. The cFA map was segmented by Atropos two-class segmentation into white matter fractions and non-white matter fractions. Similar to gray matter-based spatial statistics [23, 24], gray matter fraction was obtained by one minus the CSF and white matter fractions (Fig. 1). Pseudo-T2 images were generated by mixing the maps corresponding to gray matter and CSF fractions in 1:2 ratio. Pseudo-T2 image was generated instead of pseudo-T1 as used in a previous study [7] because the increased contrast in the CSF region may increase the accuracy of CSF segmentation. The CSF region was defined as the voxels with free water fraction larger than 0.8, and voxels being classified as subarachnoid space or ventricles by FreeSurfer segmentation of the pseudo-T2 image [25].

#### CSF parcellation

FreeSurfer segmentation already contains the masks of lateral, third and fourth ventricles. To parcellate the subarachnoid space, the sulcus-specific atlas was registered to the pseudo-T2 images using the symmetric normalization technique in ANTs [26]. To ensure the registered atlas only covered the gray matter regions, voxels outside the gray matter mask from FreeSurfer segmentation were discarded (Supplemental Method). This gray matter atlas enabled subarachnoid space parcellation using the nearest neighbor approach: For each voxel in the subarachnoid space, its label was derived from the label of the gray matter voxel that it is closest to in Euclidean distance (Figure S2). All segmentation and parcellation were performed in the native pseudo-T2 space. Parcellation results in a typical participant were shown in Figure S3.

#### Regional pseudodiffusivity

The resulting subarachnoid space and ventricular parcellation atlas was warped to the low-b dMRI images. The transform was obtained by rigidly registering the average of the dMRI volumes with b-value of 0 s/mm^2^ (b0 volume) to the b0 volume of the low-b dMRI image. For each voxel defined by the CSF parcellation atlas, apparent diffusion coefficient was calculated using the b-values smaller than or equal to 200 s/mm^2^. The reason why only b-values ≤ 200 s/mm^2^ were chosen was because in dMRI, pseudo-diffusivity, or intra-voxel incoherent motion (IVIM) effect started to appear when b ≤ 200 s/mm^2^. When b > 200 s/mm^2^, the predominant cause of signal attenuation is diffusion of water molecules [27, 28].

In this paper, only voxels with a free water fraction of 0.8 or larger were considered. The signal attenuation was assumed to arise from the pseudo-diffusivity of CSF water molecules and can be modelled as a single compartment isotropic diffusion model:

$$S\left(b\right) = S\left(0\right) {e}^{-b{D}^{*}}$$

where S(b) is the signal of a voxel at a particular b-value and ${D}^{*}$ is pseudodiffusivity. ${D}^{*}$ is therefore equivalent to the apparent diffusion coefficient.

The median pseudodiffusivity (MPD) across all voxels in each CSF region was calculated. Median was used instead of mean to reduce the impact of outliers within a CSF region, the same approach towards summarizing the diffusivity in the white matter region in previous studies [29]. For CSF regions not along the midline, such as the Sylvian fissure and central sulcus, the average MPD of the left and right regions were obtained, weighted by the volume of the corresponding regions.

### Imaging metrics

Cerebral blood flow (CBF) maps were calculated using the GE workstation. To calculate cortical CBF, the pseudo-T2 image was registered to the CBF map using rigid registration. The tissue segmentation mask previously obtained on the pseudo-T2 image was warped to the CBF. The median CBF value within the cortical gray matter region was obtained. The sulcus-specific atlas was warped to the CBF map to calculate the CBF in the gray matter region underlying the sulci and cisterns.

### Statistics

#### Cohort characteristics

The distribution of each variable was assessed using the Shapiro-Wilk’s test. For normally distributed variables, mean and standard deviation were displayed, otherwise median and interquartile range (IQR). All statistical analyses were conducted on the combined cohort of normal control and patients with memory impairment.

#### Regional variations in MPD

If a CSF region contained less than 10 voxels consistently across at least 50% of the participants, it was removed from all analyses. If the region contained less than 10 voxels in less than 50% of the participants, the MPD in those participants was labeled as “missing” but the CSF region was still included in the analysis.

#### Reproducibility of MPD

For each CSF region in each participant, the MPD was calculated by randomly selecting 95% of the voxels repeated 100 times. The coefficient of variation (standard deviation divided by the mean) was calculated.

#### Inter-regional correlation of MPD

Correlation of MPD between each pair of the CSF region was computed. Correlation *p*-values were calculated using the Pearson correlation test. *P*-values were adjusted for multiple testing by the Benjamini-Hochberg method.

Bootstrapping was used to evaluate the robustness of pairwise correlation of MPD. 95% of the cohort was randomly selected and the correlation between MPD across different brain regions was calculated. Random selection was repeated ten times. Across all the folds of random selection, pairwise correlation between each CSF region was averaged and the final *p*-value was derived using the aggregated Cauchy association test across the Pearson correlation test *p*-values.

#### Association with cognition

The association between MPD and different cognitive performance was measured using Pearson correlation and linear regression. All variables except sex were z-transformed. In measuring the association with cognitive performance (MMSE, MOCA, STM, LTM, TMTA), linear regression was corrected for age, sex and years of education. No correction was performed in associating MPD with age. Association with sex was determined by the Wilcoxon rank sum test. For each variable, multiple testing correction was performed using Benjamini-Hochberg method across all the CSF regions.

In a sensitivity analysis, the association between MPD and cognitive performance was adjusted for either intraparenchymal volume or intraparenchymal volume fraction in addition to age, sex and years of education. Intraparenchymal volume was defined as the sum of cerebral gray and white matter volumes obtained from FreeSurfer segmentation of the T1 image (FreeSurfer labels 2, 3, 41 and 42). Intraparenchymal volume fraction was defined as the intraparenchymal volume divided by intracranial volume, which was obtained using the sienax function in FSL [30].

#### Fine mapping near the third ventricle

Lateral and third ventricles were subdivided into four zones with respect to the start of the third ventricle (Figure S4). This region was identified by dilating the lateral ventricular masks by 3 voxels in the pseudo-T2 image and finding the overlap between the dilated mask and the third ventricle (Figure S5). For lateral ventricles, zone 1 was defined as the region between 15 and 20 mm away from the start of the third ventricle, zone 2 between 10 and 15 mm, zone 3 between 5 and 10 mm and zone 4 less than 5 mm. For the third ventricle, zone 1 was the region within 5 mm away from the start of the third ventricle, zone 2 between 5 and 10 mm, zone 3 between 10 and 15 mm, and zone 4 between 15 and 20 mm. Areas that were more than 20 mm away from the start of the third ventricle were not analyzed. MPD was obtained in each zone and associated with cognitive performance using linear regression corrected for age, sex and years of education and adjusted for multiple testing across all zones using Benjamini-Hochberg method.

#### Association with imaging markers

Linear regression was performed between the MPD of each sulcus and cortical CBF. For regional CBF, linear regression was performed between the MPD of each sulcus and the CBF of the gray matter region that underlies the corresponding sulcus. All regression analyses were corrected for age and sex. All variables except sex were z-transformed. For each variable, multiple testing correction was performed using Benjamini-Hochberg method across all the CSF regions. Average CBF map across the entire cohort was shown in Figure S6.

#### Softwares

The CBSS algorithm was implemented in Python 3.9 and Fortran 90. All statistical analyses were performed in R 4.1.0. FreeSurfer version 7.4.0 and FSL version 6.0.7.2 were used. All analyses were performed on Centos Linux 7.9 using Intel Xeon CPUs. The source code is published: https://github.com/Yutong441/CBSS.

## Results

### Cohort characteristics

Ninety-four participants were recruited. One participant had significant motion artifacts in the low-b dMRI scan and was excluded from the analysis. In total, 58 patients (age 70.7 [± 7.9] years, 36.2% male) and 35 controls (age 63.7 [± 9.0] years, 40% male) were included in the study (Table 1).

**Table 1:** Demographic features

Feature	Total	Cognitively impaired	Normal control	*p*
Number	93	58	35	
Age (years), mean ± std	68.1 ± 9.0	70.7 ± 7.9	63.7 ± 9.0	< 0.001
Educations (years), median (IQR)	12.0 (9.0–15.0)	10.5 (8.3–12.8)	13.0 (9.0–16.0)	0.057
Sex (male), *n* (%)	36 (38.3)	21 (36.2)	14 (40.0)	0.885
Cortical CBF (mL/100 g/min), mean ± std	48.7 ± 14.0	45.8 ± 12.9	53.6 ± 14.7	0.012
MMSE, median (IQR)	26.0 (21.0–28.0)	22.5 (19.0–26.0)	28.0 (28.0–30.0)	< 0.001
MOCA, median (IQR)	20.0 (15.0–26.0)	16.5 ± 6.0	26.5 (23.2–28.0)	< 0.001

*p*-values between cognitively impaired and normal control participants were shown

CBF, Cerebral blood flow; MMSE, Mini-mental state examination; MOCA, Montreal cognitive assessment

### Regional variations in MPD

**Fig. 2:**
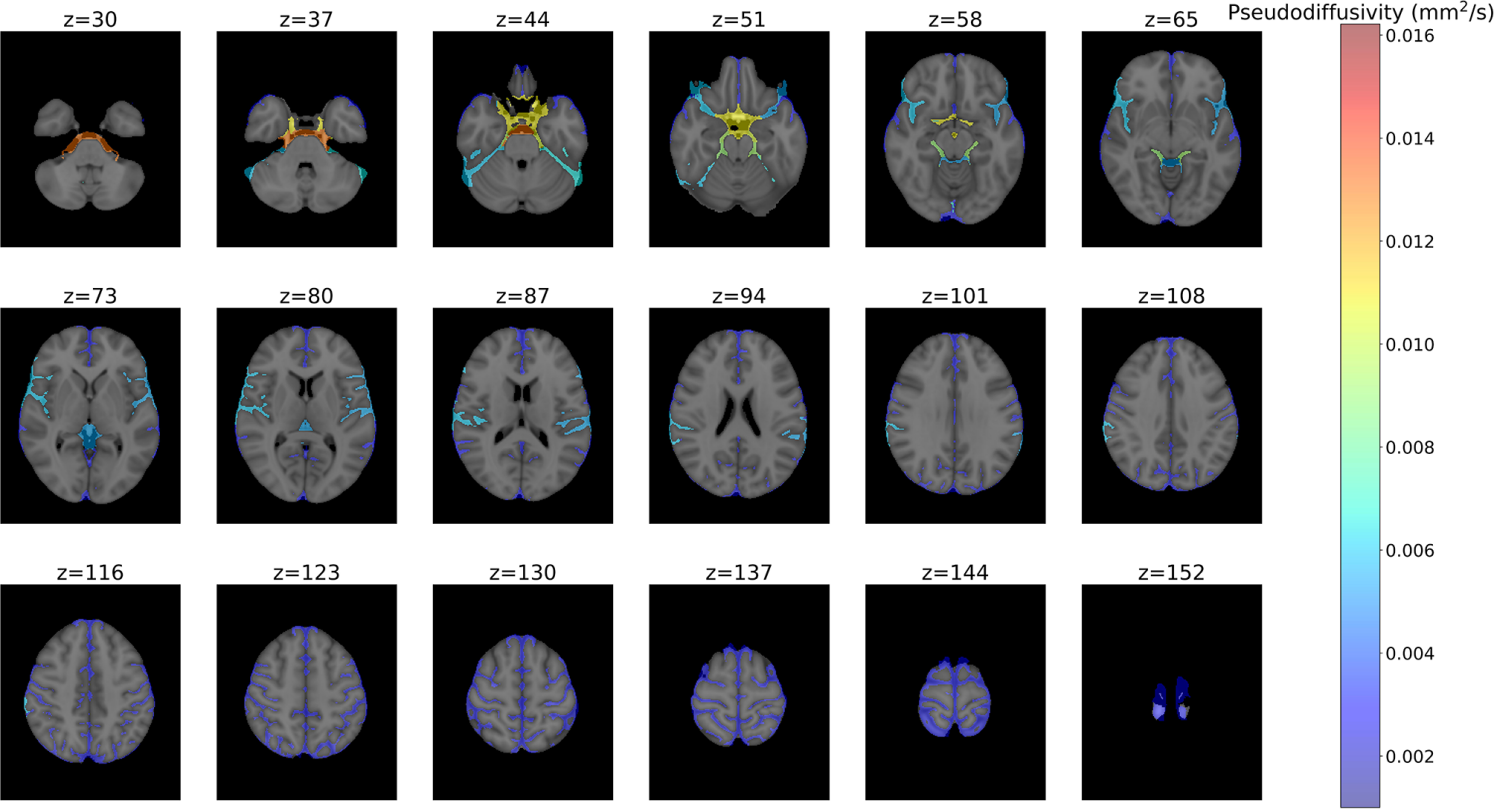
The mean of median pseudodiffusivity (in mm^2^/s) in each CSF region across all participants

Across the 93 participants, among the 19 CSF regions included in this study, cerebellomedullary cisterns and premedullary cisterns were not captured in the 96.8% and 75.3% of the low-b dMRI scans and these two regions were excluded from the analysis. On average, the regions with the highest MPD were the prepontine cistern, suprasellar cistern, third and fourth ventricles. The regions with the lowest MPD were the precentral, central, postcentral and intraparietal sulci (Fig. 2, Figure S7). Regions with higher MPD also demonstrated higher variability in MPD.

### Reproducibility of MPD

To evaluate the reproducibility of MPD, we used bootstrapping to calculate the coefficient of variation of each CSF region in each participant. In most regions, the coefficient of variation did not exceed 0.05 (Figure S8). Regions with the highest coefficients of variation were the cerebellopontine angle cistern, crural cistern, third ventricle, fourth ventricle and superior temporal sulcus, which had smaller volumes (Figure S9).

### Inter-regional correlation of MPD

**Fig. 3:**
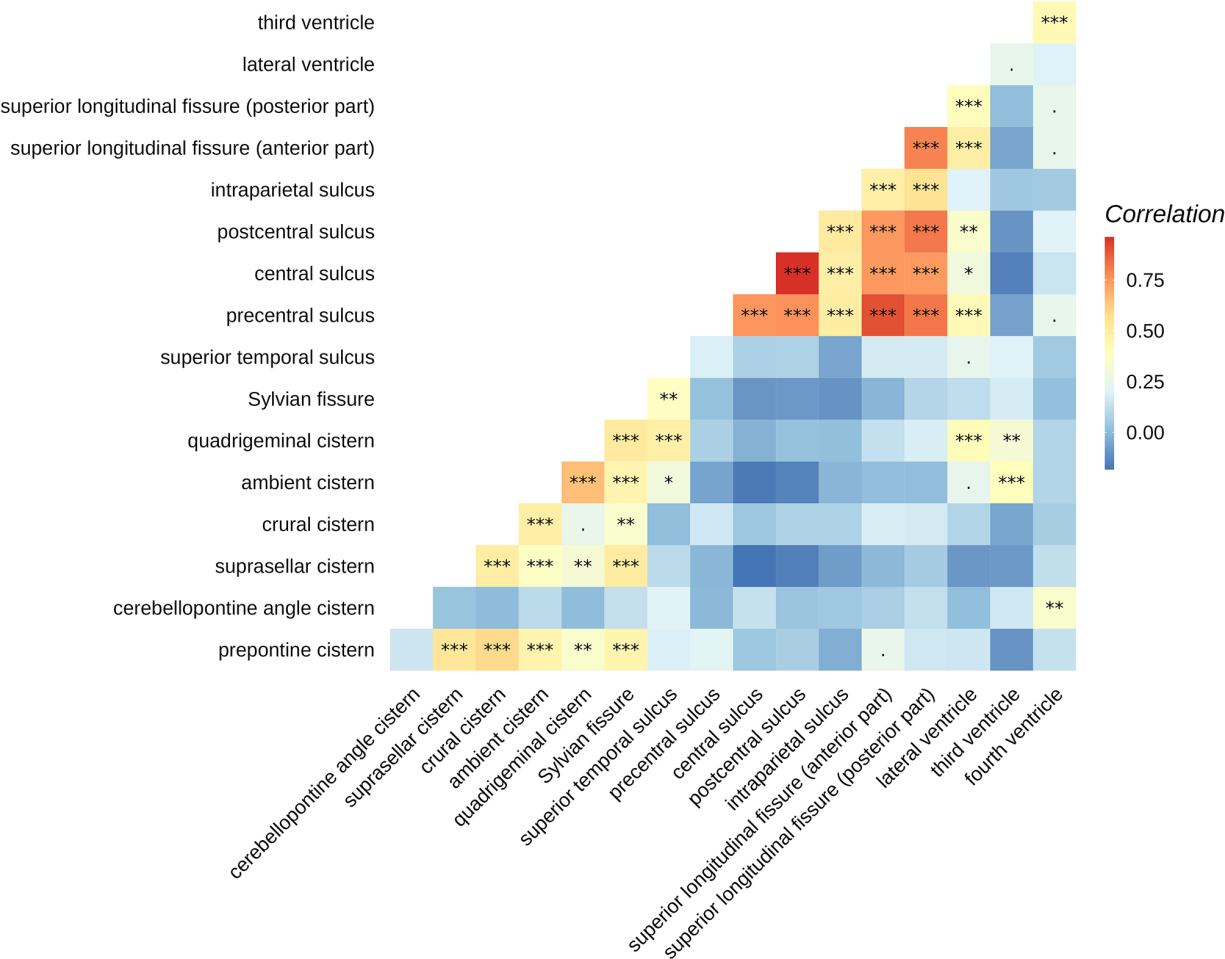
Correlation of pseudodiffusivity between different CSF regions. *P* values were labelled as: ***: <0.001, **: 0.001–0.01, *: 0.01–0.05, .: 0.05–0.1

We found two groups of regions whose MPD profiles were correlated with each other. One group occurred near the cerebral arteries: prepontine cistern, suprasellar cistern, crural cistern, ambient cistern and Sylvian cistern. The other group was away from the arteries: the precentral, central, postcentral, intraparietal sulcus, superior longitudinal fissure, and the lateral ventricle. The MPDs in the third ventricle and cerebellopontine angle cistern were not significantly associated with most CSF regions (Fig. 3). Statistical significance of these associations remained unchanged in the bootstrapping analysis (Figure S10).

### Association with demographics and cognition

**Fig. 4:**
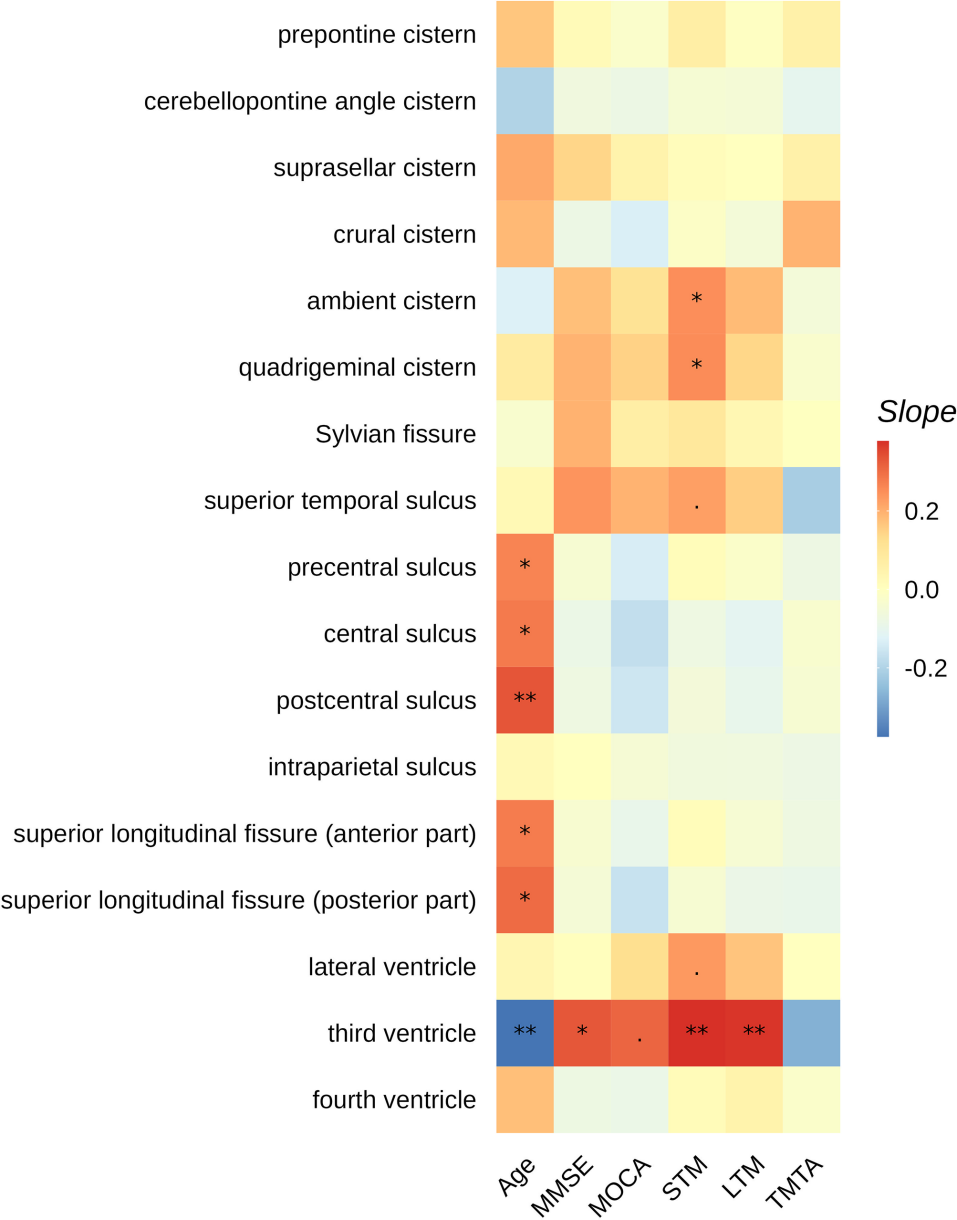
Linear regression slopes between MPD in different CSF regions with cognitive performance corrected for age, sex and years of education. *P* values were labelled as: ***: <0.001, **: 0.001–0.01, *: 0.01–0.05, .: 0.05–0.1. Higher scores in MMSE, MOCA, STM and LTM indicate better cognitive performance. Shorter TMTA time indicates better processing speed. Abbreviations: MMSE: mini-mental state examination, MOCA: Montreal cognitive assessment, STM: short term memory, LTM: long term memory, TMTA: trail making time A

Across the entire cohort, the MPDs in the cluster of CSF regions away from the cerebral arteries demonstrated positive association with age (postcentral sulcus *R* = 0.34, slope = 0.33, adjusted *p* = 0.007; central sulcus *R* = 0.28, slope = 0.28, adjusted *p* = 0.021; precentral sulcus *R* = 0.28, slope = 0.27, adjusted *p* = 0.025; anterior part of the superior longitudinal fasciculus *R* = 0.29, slope = 0.28, adjusted *p* = 0.021; posterior part of the superior longitudinal fasciculus *R* = 0.31, slope = 0.30, adjusted *p* = 0.016) (Fig. 4). Third ventricle MPD was negatively associated with age (*R*=-0.40, slope=-0.38, adjusted *p* < 0.001). No CSF regions displayed significant sex-specific differences in MPD. For cognitive performance, the third ventricle MPD was positively associated with MMSE (*R* = 0.39, slope = 0.31, adjusted *p* = 0.025), STM (*R* = 0.44, slope = 0.38, adjusted *p* = 0.005) and LTM (*R* = 0.46, slope = 0.37, adjusted *p* = 0.005) (Fig. 4, Figure S11). After correcting for intraparenchymal volume, the third ventricle MPD was positively associated with STM (*R* = 0.44, slope = 0.35, adjusted *p* = 0.018) and LTM (*R* = 0.46, slope = 0.34, adjusted *p* = 0.024), but not MMSE (*R* = 0.39, slope = 0.26, adjusted *p* = 0.138) (Figure S12). A similar trend was found after correcting for intraparenchymal volume fraction (Figure S13) (STM: *R* = 0.44, slope = 0.35, adjusted *p* = 0.015; LTM: *R* = 0.46, slope = 0.35, adjusted *p* = 0.017; MMSE: *R* = 0.39, slope = 0.29, adjusted *p* = 0.173). The MPD in the quadrigeminal and ambient cisterns were positively associated with STM (ambient: *R* = 0.29, slope = 0.25, adjusted *p* = 0.048, quadrigeminal: *R* = 0.28, slope = 0.25, adjusted *p* = 0.048). For the CSF regions away from the cerebral arteries, there was insignificant negative association with cognitive performance (Fig. 4).

### Fine mapping near the third ventricle

**Fig. 5:**
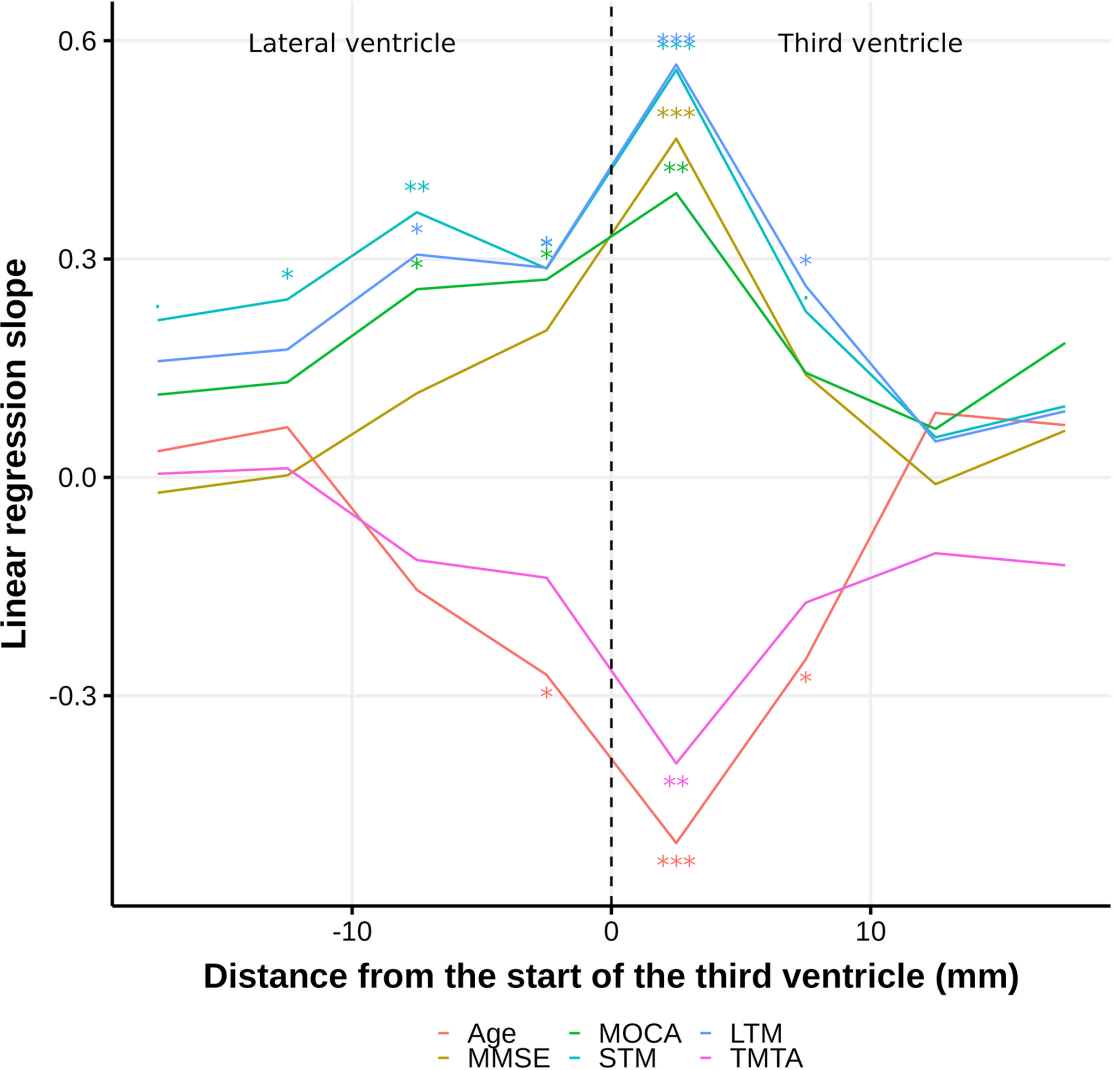
Linear regression slopes between different variables and the MPD in ventricular regions with different distances from the start of the third ventricle, adjusted for age, sex and years of education. *P* values were labelled as: ***: <0.001, **: 0.001–0.01, *: 0.01–0.05, .: 0.05–0.1. Abbreviations: MOCA: Montreal cognitive assessment, MMSE: mini-mental state examination, STM: short term memory, LTM: long term memory, TMTA: trail making test A

The ventricular region most strongly associated with age and cognitive performance was within 5 mm from the start of the third ventricle (Fig. 5). The MPD in this region was associated with age (slope=-0.51, adjusted *p* < 0.001), MMSE (*R* = 0.46, slope = 0.45, adjusted *p* = 0.002), MOCA (*R* = 0.45, slope = 0.37, adjusted *p* = 0.020), STM (*R* = 0.53, slope = 0.54, adjusted *p* < 0.001), LTM (*R* = 0.59, slope = 0.54, adjusted *p* < 0.001) and TMTA (*R*=-0.46, slope=-0.37, adjusted *p* = 0.020). The significant association persisted within 10 mm from the foramen both in the third and lateral ventricles.

### Association with imaging markers

**Fig. 6:**
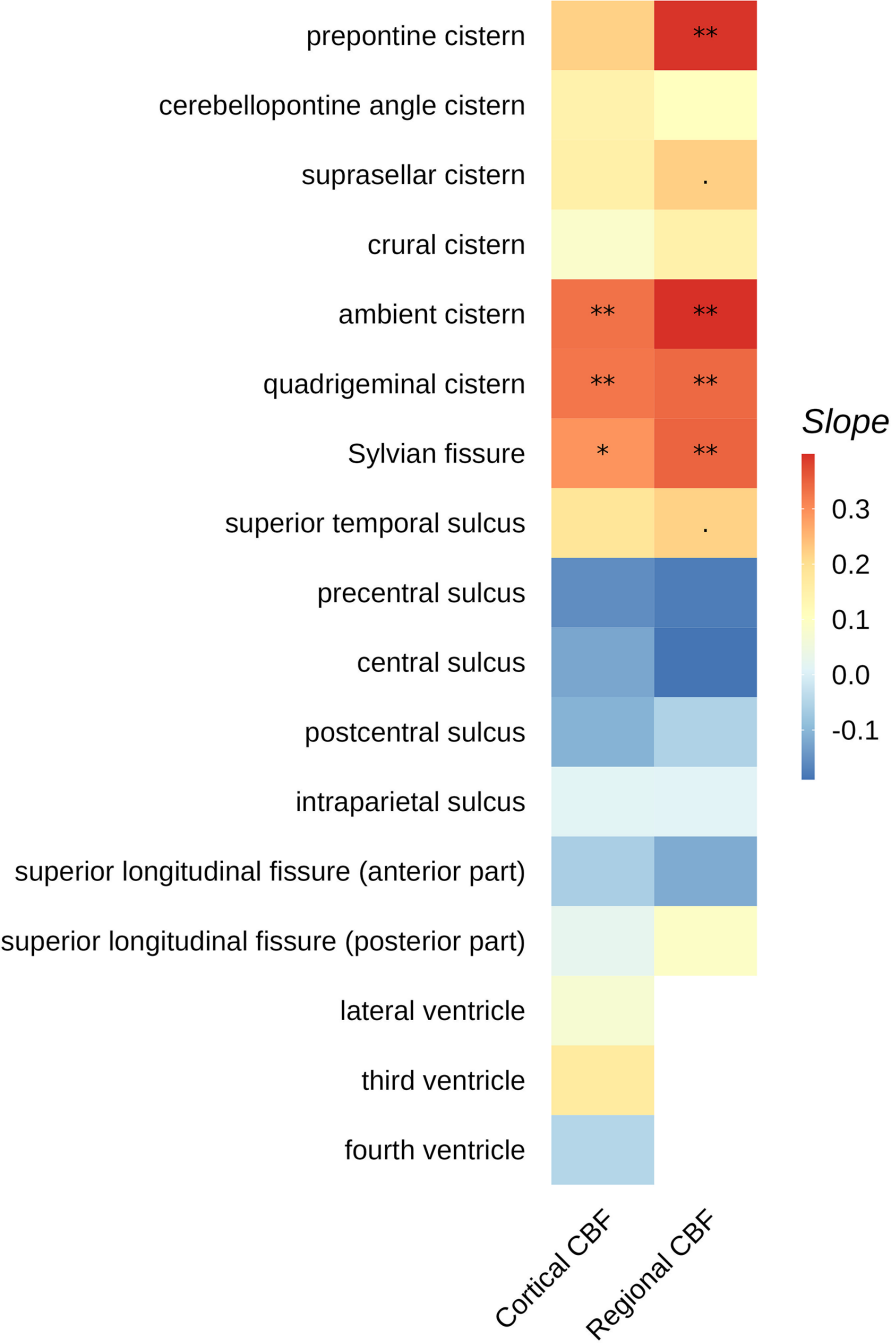
Linear regression coefficients between median pseudodiffusivity in different CSF regions with different imaging biomarkers, corrected for age and sex and adjusted for multiple testing. Cortical CBF is the median cerebral blood flow (CBF) in the cortical gray matter. Regional CBF is the median CBF in the gray matter region that underlies a particular sulcus or cistern

The MPD in the ambient, quadrigeminal cisterns and Sylvian fissure was significantly associated with cortical CBF (ambient: *R* = 0.39, slope = 0.33, adjusted *p* = 0.005; quadrigeminal: *R* = 0.30, slope = 0.33, adjusted *p* = 0.005; Sylvian: *R* = 0.31, slope = 0.29, adjusted *p* = 0.012) (Fig. 6). For ambient, quadrigeminal, prepontine cisterns and Sylvian fissure, the MPD was significantly associated with the CBF in the gray matter area underlying the cisterns/sulci (ambient: *R* = 0.41, slope = 0.40, adjusted *p* = 0.001; quadrigeminal: *R* = 0.30, slope = 0.34, adjusted *p* = 0.002; prepontine: *R* = 0.36, slope = 0.40, adjusted *p* = 0.001; Sylvian: *R* = 0.34, slope = 0.35, adjusted *p* = 0.001). We selected the CSF regions that were significantly associated with regional CBF before multiple testing correction: ambient, quadrigeminal, suprasellar, prepontine cisterns, Sylvian fissure and superior temporal sulcus. Among these six regions, the MPD-CBF correlation in each participant increased with aging (*R* = 0.38, *p* < 0.001).

## Discussion

We have developed a novel technique to quantify CSF pseudodiffusivity in the major sulci, cisterns and ventricles. In a cohort of 93 participants with varying degrees of cognitive impairment, we have shown the pseudodiffusivity in the third ventricle was significantly associated with both short and long term memory. Third ventricle pseudodiffusivity could represent a novel imaging biomarker of cognition, corroborating the role of CBSS in unbiased, quantitative interrogation of the clinical implications of CSF pseudodiffusivity.

CBSS is automatic, easy to implement and interpret. It uses gray matter as landmarks to identify CSF regions. By leveraging registration algorithms that are well established in cortical parcellation, CBSS eliminates the need to manually define regions of interest to study regional CSF motion as in previous studies [6, 31, 32]. While a similar quantitative technique has been proposed recently [7], this method divided CSF regions based on pseudodiffusivity patterns across the cohort rather than pre-defined anatomical boundaries based on CSF flow covariance. The anatomically-oriented approach in CBSS allows for more fine grained analysis of CSF flow in the extra-axial space adjacent to the cortical structures.

Measuring CSF pseudodiffusivity is susceptible to partial volume effects from adjacent gray matter regions. To reduce the partial volume effects, we only consider voxels with a CSF fraction above 0.8. The robustness of the technique is shown as correlation of third ventricle MPD with cognitive performance persisted after correcting for intraparenchymal volume. We obtained pseudodiffusivity patterns consistent with previous studies, as the subarachnoid spaces near cerebral arteries have higher pseudodiffusivity compared with those towards the cortex [6, 7]. Third ventricle pseudodiffusivity negatively correlated with age [6], while the pseudodiffusivity in the cortical sulci positively correlated with age [7, 32]. These results suggest that CBSS is a robust technique of studying regional CSF pseudodiffusivity.

CBSS is a region-wise, rather than voxel-wise, analysis approach. Summarizing voxel statistics within each region can confer robust results given the low signal-to-noise ratio and low resolution in low-b dMRI imaging [33]. However, this region-wise approach assumes that patterns of pseudodiffusivity follow anatomical boundaries. This assumption could be justified as a previous study found different pseudodiffusivity covariance patterns tend to represent distinct physiologic drivers of CSF flow and compartmentalization of the CSF spaces by arachnoid membranes and brain parenchyma [7].

Using CBSS, we identified two main groups of sulci with similar MPD profiles. One group–Sylvian fissure, quadrigeminal cistern, ambient cistern, crural cistern, suprasellar cistern and superior temporal sulcus–exhibited higher MPD and are located in the vicinity of large arteries (Figure S14). The other group–central sulcus, precentral sulcus, postcentral sulcus, intraparietal sulcus and superior longitudinal fissure–exhibited lower MPD and are located away from large arteries. One explanation could be that arterial pulsation enhances local CSF flow. Future studies could apply CBSS to investigate whether local CSF pseudodiffusivity is reduced in patients with reduced pulsatility of large vessels such as intracranial stenosis.

We have conducted the first study to dissect the relationship between regional CSF pseudodiffusivity and cognitive performance. MPD in the third ventricle was positively associated with short and long term memories, and less strongly with processing speed (TMTA) and general cognition (MOCA). This is consistent that in AD mice models with overexpression of human amyloid precursor protein, a lower CSF flow in the third ventricle was reported compared with wild type mice [34, 35]. Future research could investigate why the pseudodiffusivity in the third ventricle correlates with cognition better than other CSF regions in humans.

CBSS has revealed the spatial patterns of MPD-CBF association. We found that the MPD in the sulci/cisterns near the large arteries were linked to higher CBF in the adjacent cortical regions, including the Sylvian fissure (contain the middle cerebral artery), ambient and quadrigeminal cisterns (contain the posterior cerebral artery) and prepontine cisterns (contain the basilar artery). The MPD-CBF association could be mediated by arterial pulsatility [36], cardiac function [37] and/or neurovascular coupling [38]. By causing distension and relaxation of blood vessels as a result of cardiac cycles or neuronal activities, all three mechanisms alter intracranial blood volume and drive CSF flow according to Monro-Kellie doctrine [9, 39]. Therefore, in conjunction with CBSS, future research could employ 4D flow phase contrast MRI and near infrared spectroscopy [38] to disentangle the contribution of arterial/cardiac pulsations and neurovascular coupling to CSF pseudodiffusivity.

There are limitations that warrant discussion. Firstly, we found no sex-specific difference in pseudodiffusivity, in contrast to a previous study showing that females exhibit higher pseudodiffusivity in certain CSF regions [7]. The lack of significant differences could be attributed to the high proportion of females in our cohort that may bias the results. Secondly, the participants from memory clinics received clinical diagnoses of AD or mild cognitive impairment but the diagnoses were not confirmed by AD biomarkers such as amyloid PET. Future research could investigate the relationship between AD biomarkers and CSF pseudodiffusivity. Thirdly, this study acquired single-shell dMRI from which free water fraction was derived as a proxy of CSF fraction. However, the single-shell-based free water model may not be able to distinguish between free water changes and changes in tissue diffusivity [40]. Future studies should consider using multi-shell dMRI to obtain the map of CSF fraction. Fourthly, susceptibility-induced distortion in dMRI was not corrected due to the lack of reverse phase encoding direction images in this study. Future studies should investigate the impact of susceptibility-induced distortion on pseudoffusivity values.

## Conclusion

In conclusion, we have developed a novel technique of quantifying regional CSF pseudodiffusivity that is automatic and robust in low resolution low-b dMRI images. Using CBSS, we have discovered the pseudodiffusivity in the third ventricle to be a potential biomarker of impaired memory and cognition.

### Electronic supplementary material

Below is the link to the electronic supplementary material.

Supplementary Material 1

## Data Availability

The imaging and cognitive data in this study may be shared with researchers upon reasonable requests to the corresponding author.

## Abbreviations

AD: Alzheimer’s disease
CBF: Cerebral blood flow
CBSS: Cerebrospinal fluid-based spatial statistics
CDR: Clinical Dementia Rating scale
cFA: Free water corrected fractional anisotropy
CSF: Cerebrospinal fluid
LTM: Long term memory
Low-b dMRI: Low b-value diffusion magnetic resonance imaging
MCI: Mild cognitive impairment
MMSE: Mini-mental state examination
MOCA: Montreal cognitive assessment
MPD: Median pseudo-diffusivity
STM: Short term memory
TE: Echo time
TMTA: Trail making test A
TR: Repetition time
