# Supplementary material for: Cerebrospinal fluid-based spatial statistics: towards quantitative analysis of cerebrospinal fluid pseudodiffusivity
Source: Fluids Barriers CNS. 2024 Jul 18;21:59. doi: 10.1186/s12987-024-00559-z (PMC11256588; doi:10.1186/s12987-024-00559-z)
Supplement: Supplementary file 1 — Supplementary Material 1 [file 12987_2024_559_MOESM1_ESM.docx]

# Supplementary File

## Supplemental Method

To ensure the atlas registered from MNI space to the individual pseudo-T2 image only covered the gray matter region, the gray matter mask was obtained from freesurfer segmentation of the pseudo-T2 image. The label of the gray matter voxels is derived from the label of the voxel in the registered atlas that it is closest to in Euclidean space. If a gray matter voxel is more than 3mm away from the nearest voxel in the registered atlas with a label, the gray matter voxel was not labelled.

## Supplementary Figures


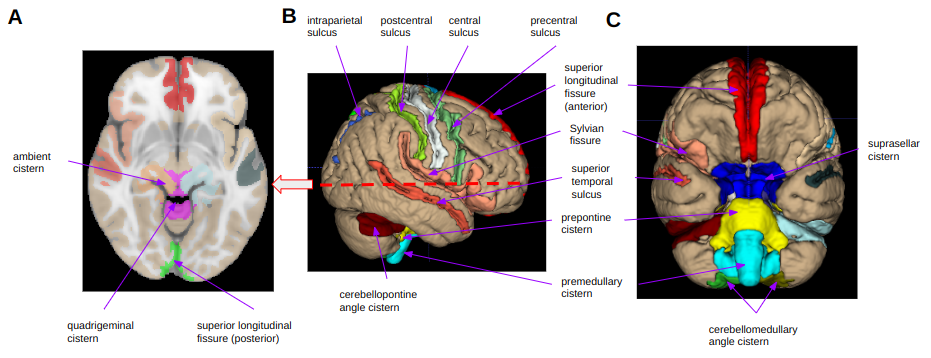


**Figure S1**. 3D view of the atlas with cortical regions labelled according to the proximity to specific sulci or cisterns. **A**: axial slice at the level of the red line in B showing the brainstem cisterns, **B**: lateral view of the surface reconstruction, **C**: anterior view of the surface reconstruction.


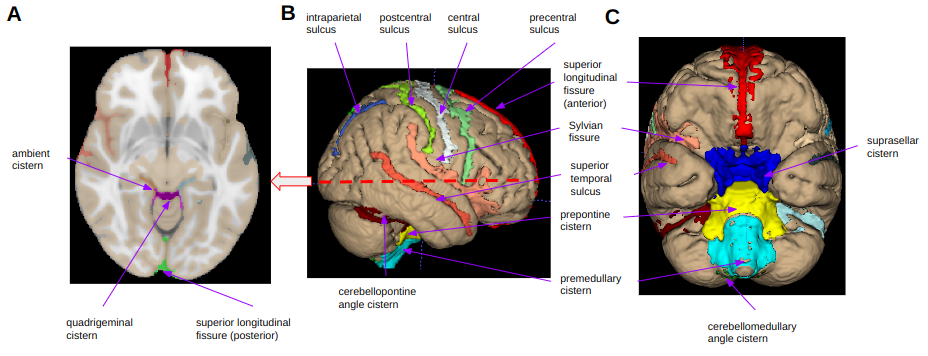


**Figure S2**. 3D view of the sulci and cisterns identified using the atlas in Figure S1. **A**: axial slice at the level of the red line in B showing the brainstem cisterns, **B**: lateral view of the surface reconstruction, **C**: anterior view of the surface reconstruction.


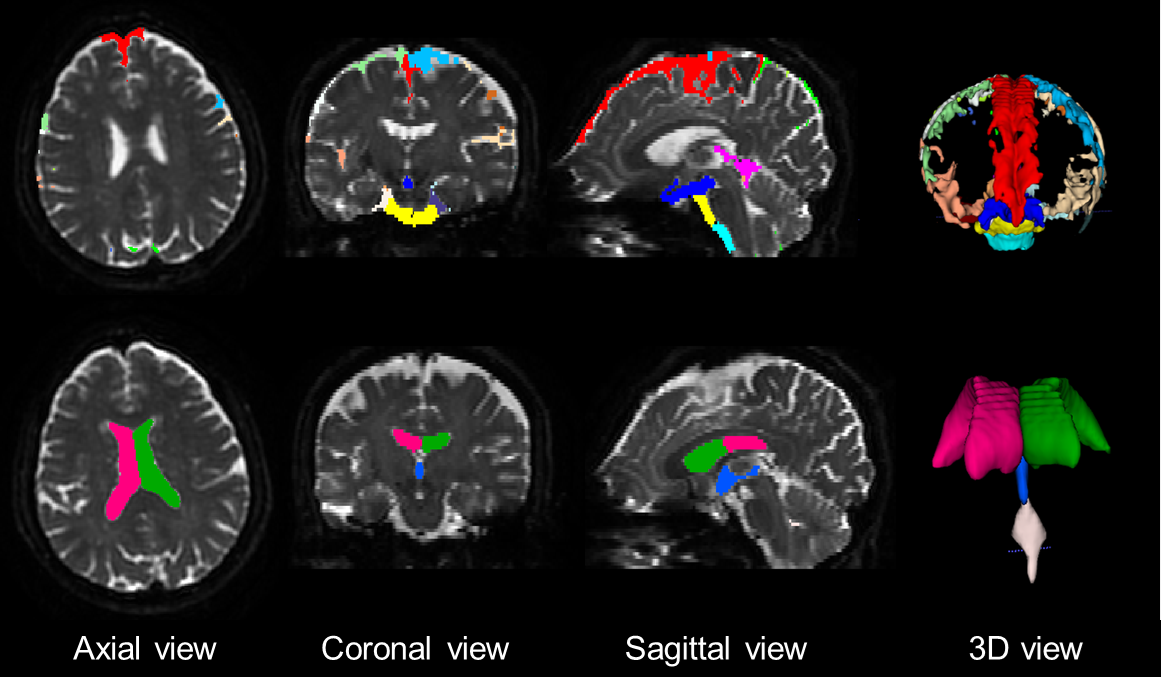


**Figure S3**. Native space ventricle segmentation and subarachnoid space parcellation overlaid with the b0 image in a typical participant.


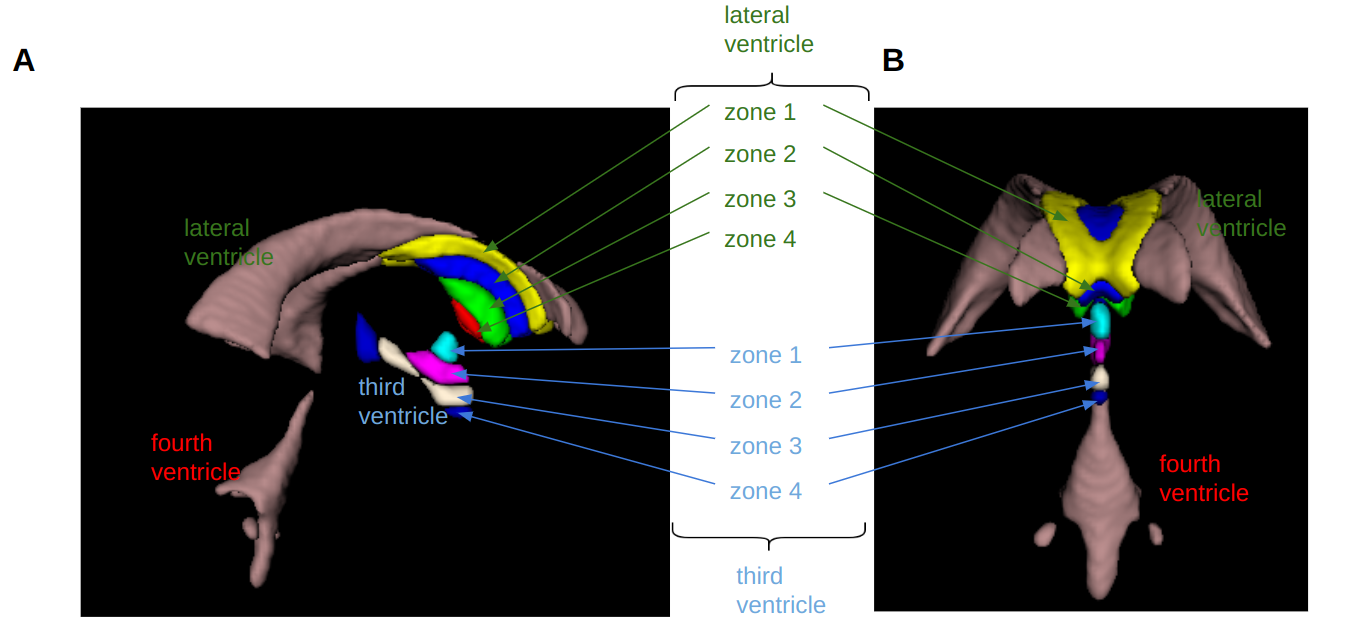


**Figure S4**. Division of ventricles into different zones.

**
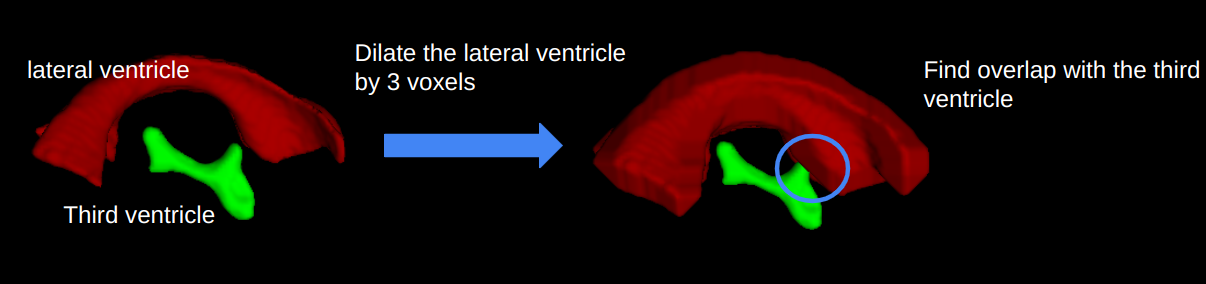
**

**Figure S5**. Identifying the start of the third ventricle.


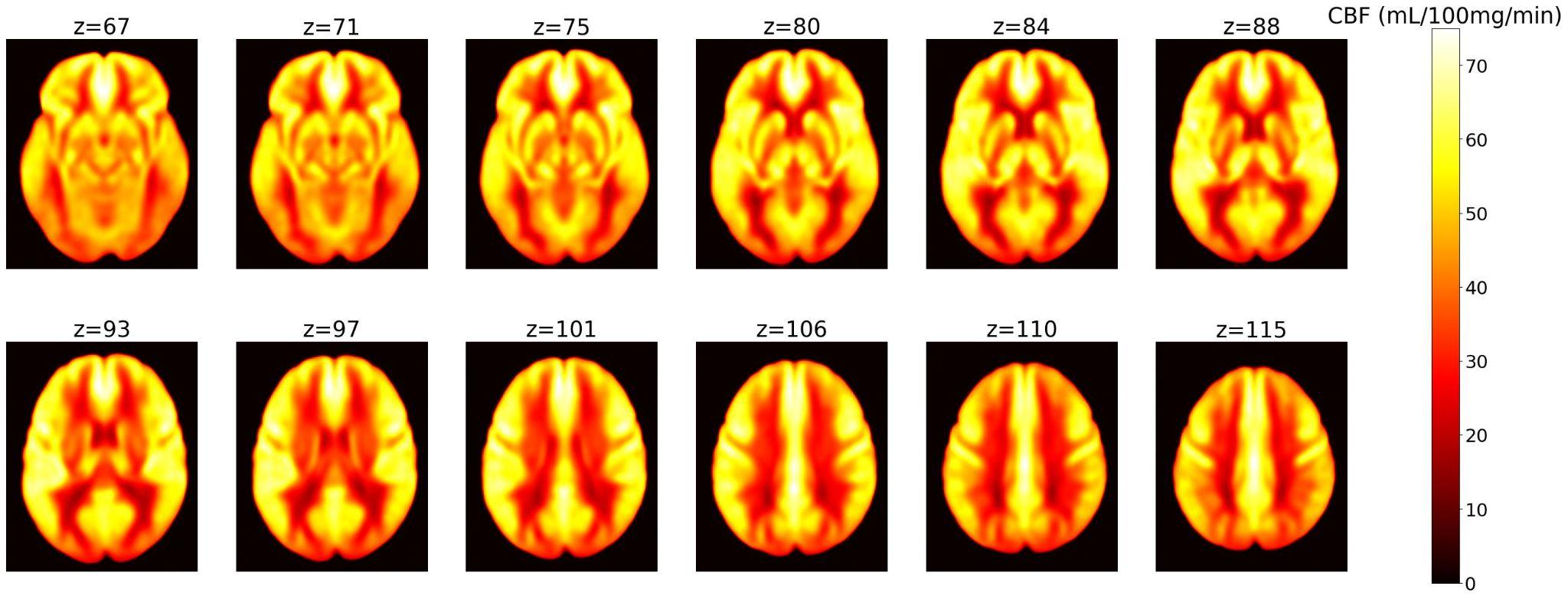


**Figure S6**: Average cerebral blood flow (CBF) map across the entire cohort in the MNI space. The CBF map of each patient was registered to their respective T1 images and then warped to the MNI space.


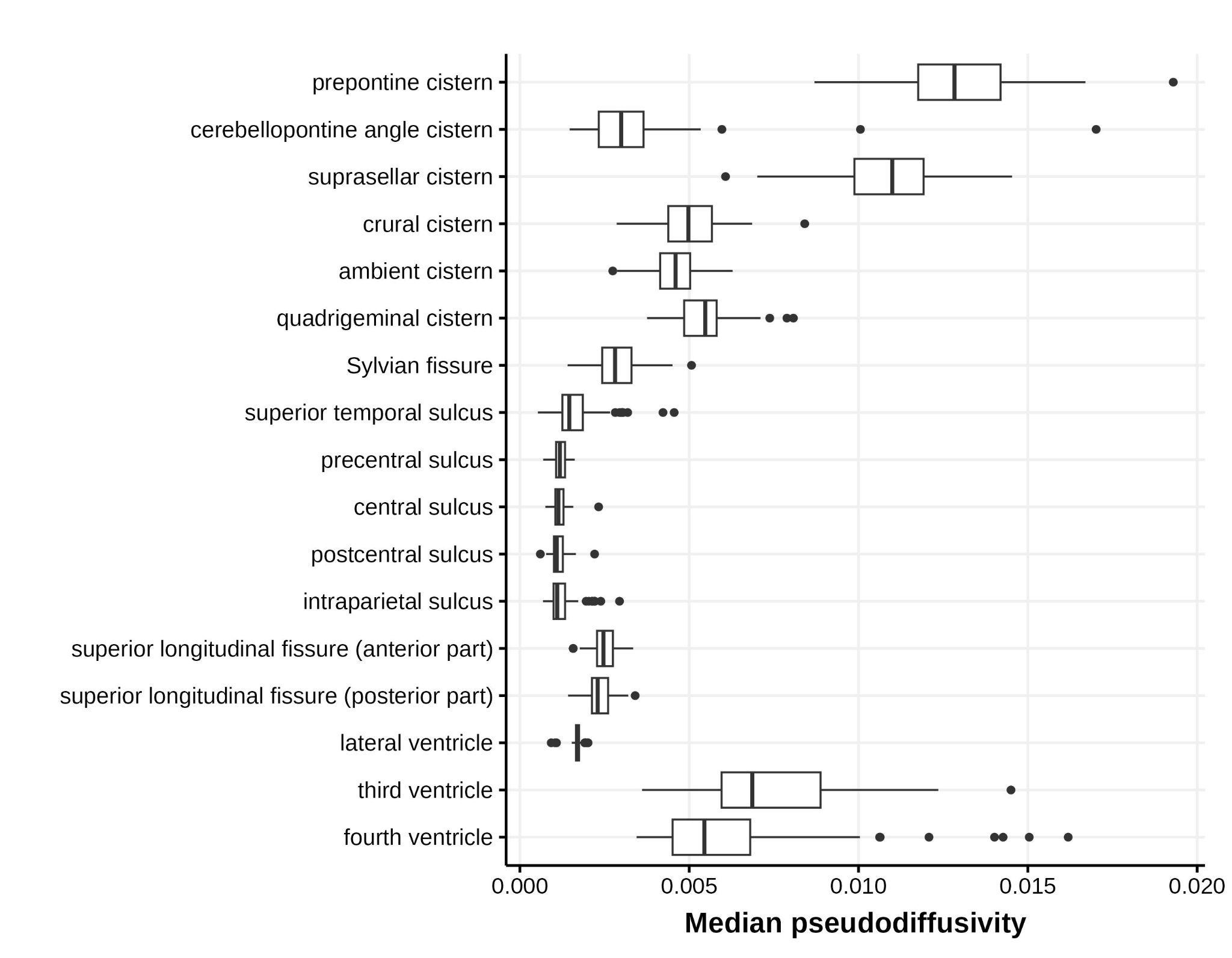


**Figure S7**. MPD in each CSF region.


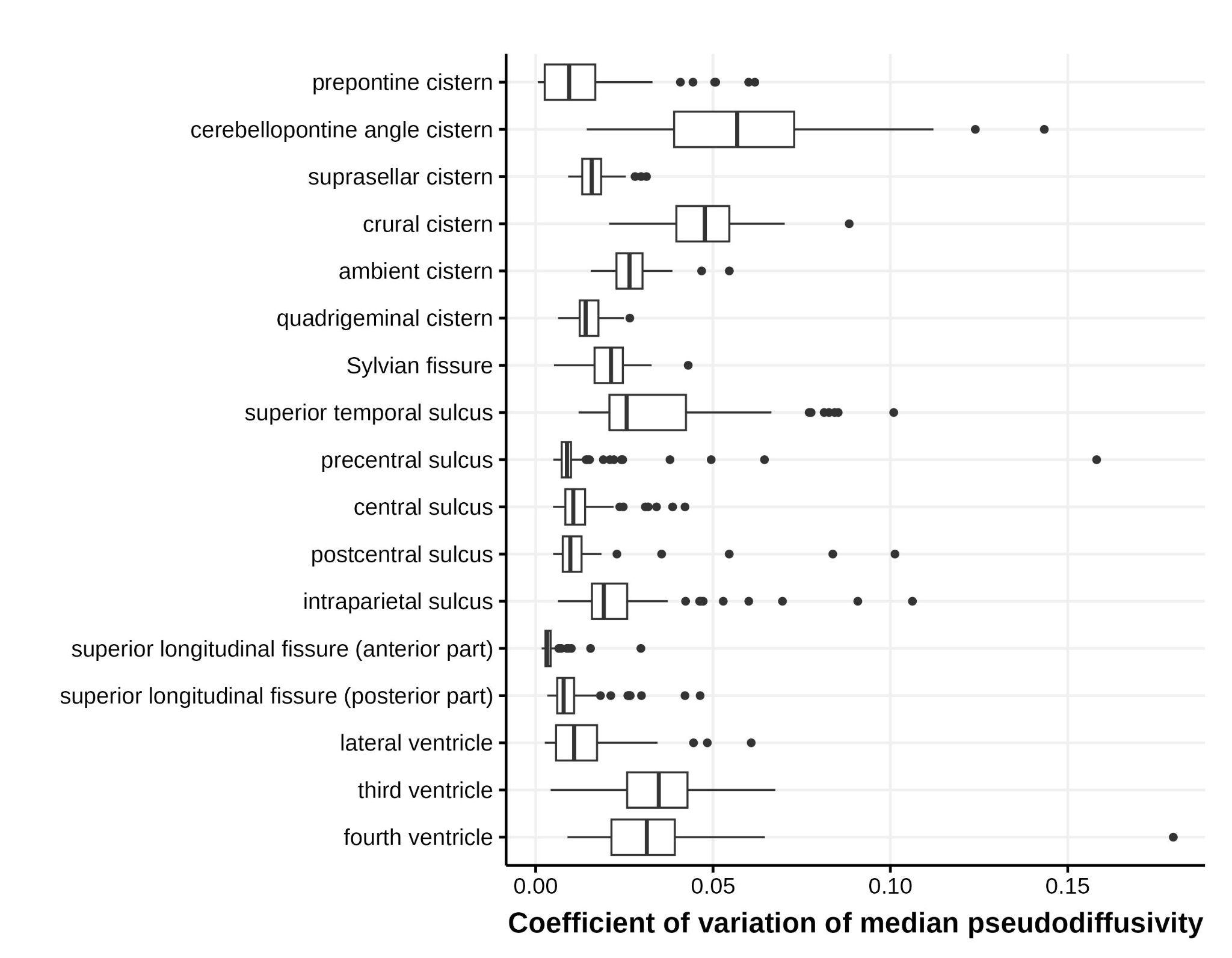


**Figure S8**. Coefficient of variation of MPD in each CSF region.


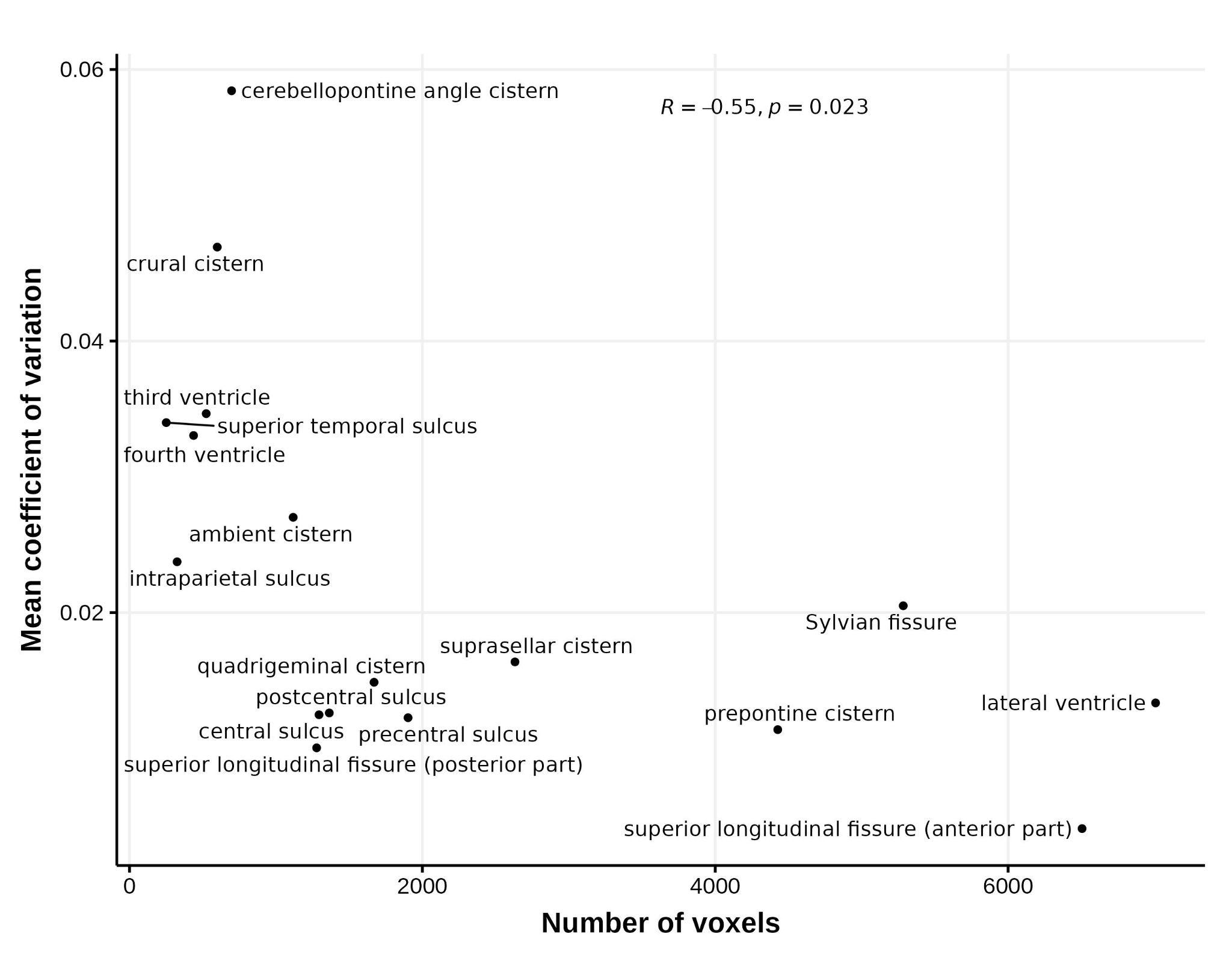


**Figure S9**. Relationship between the mean coefficient of variation of MPD and the number of voxels in each CSF region.


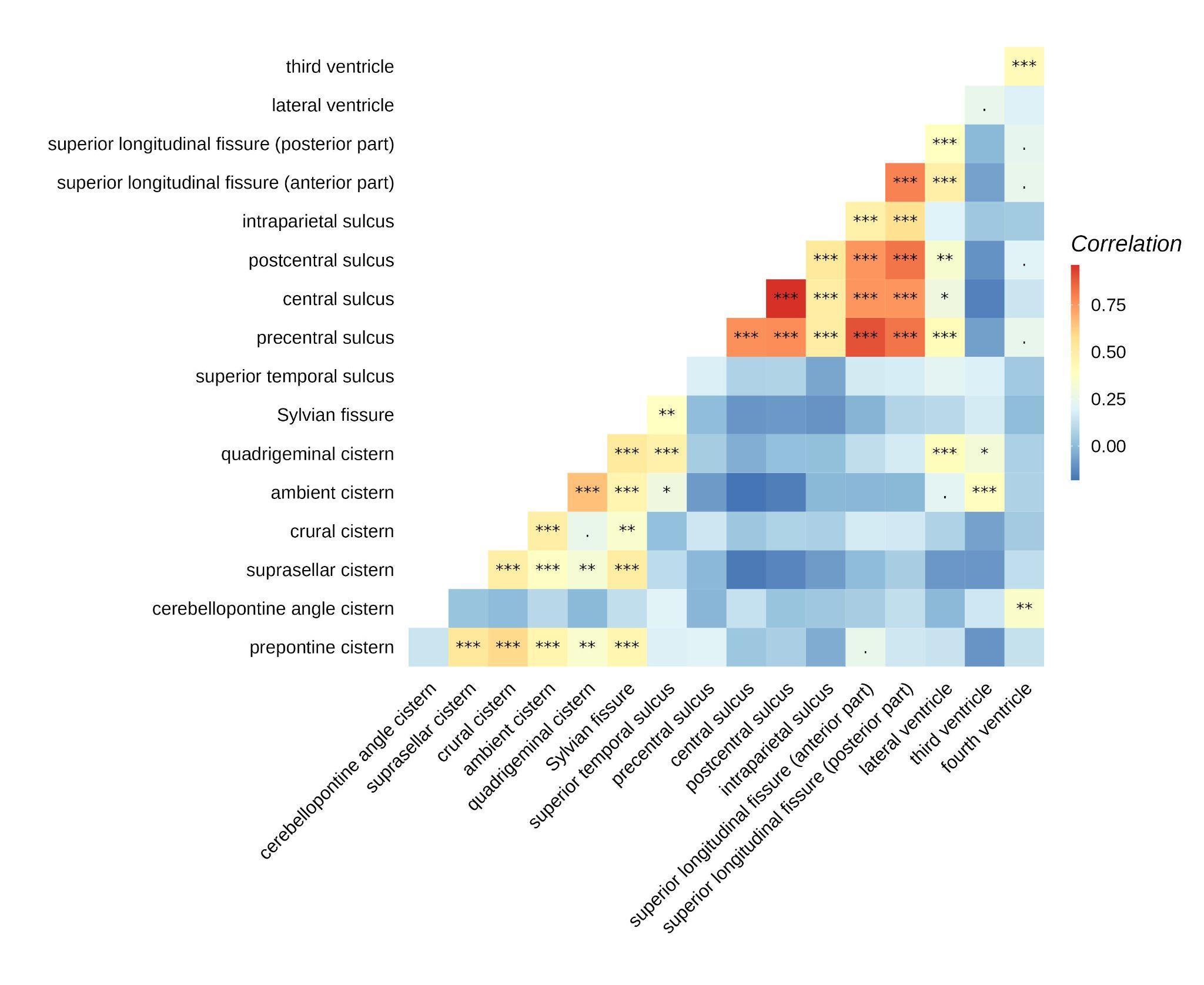


**Figure S10**. Bootstrapped correlation of MPD between different CSF regions. P values were labelled as: ***: <0.001, **: 0.001-0.01, *: 0.01-0.05, .: 0.05-0.1.


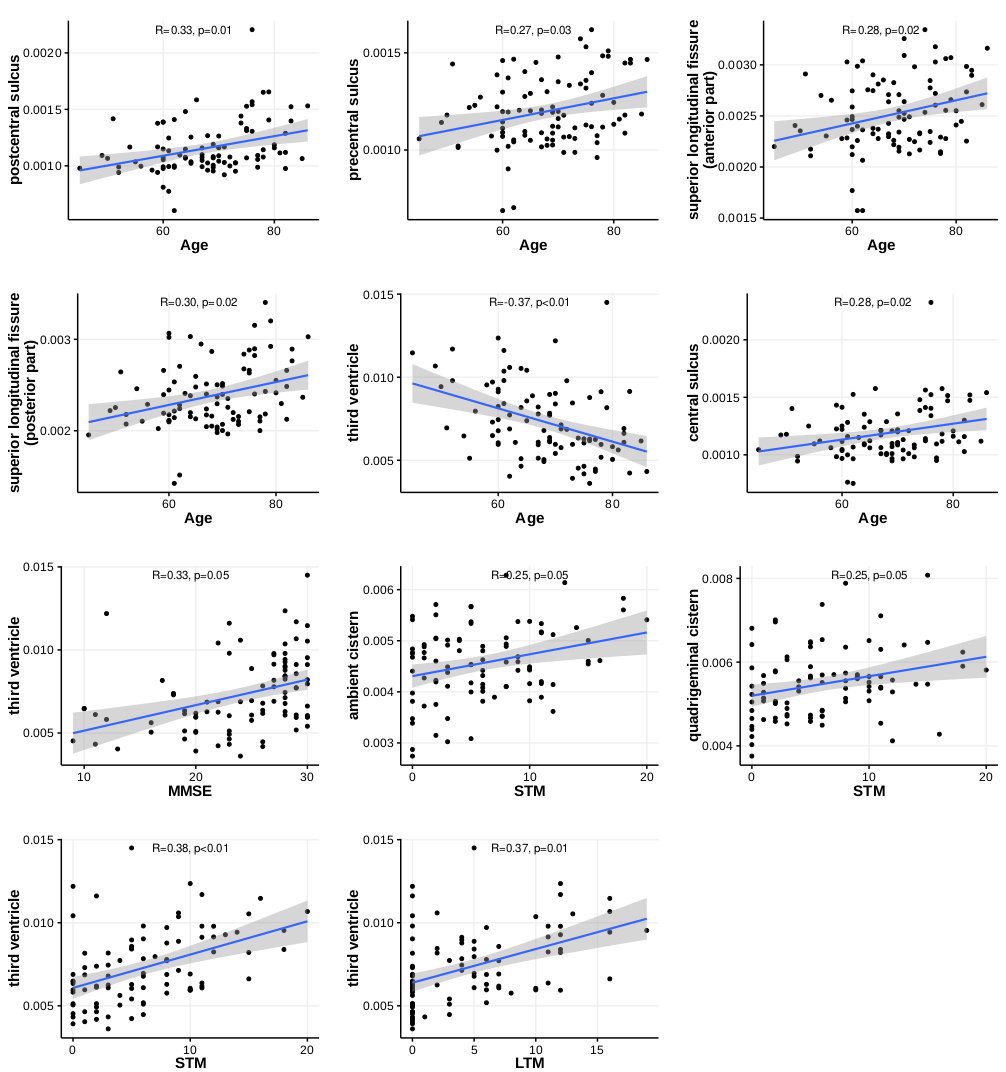


**Figure S11**. Correlation between MPD in different CSF regions and demographic and cognitive variables. P-values adjusted for multiple testing were shown.


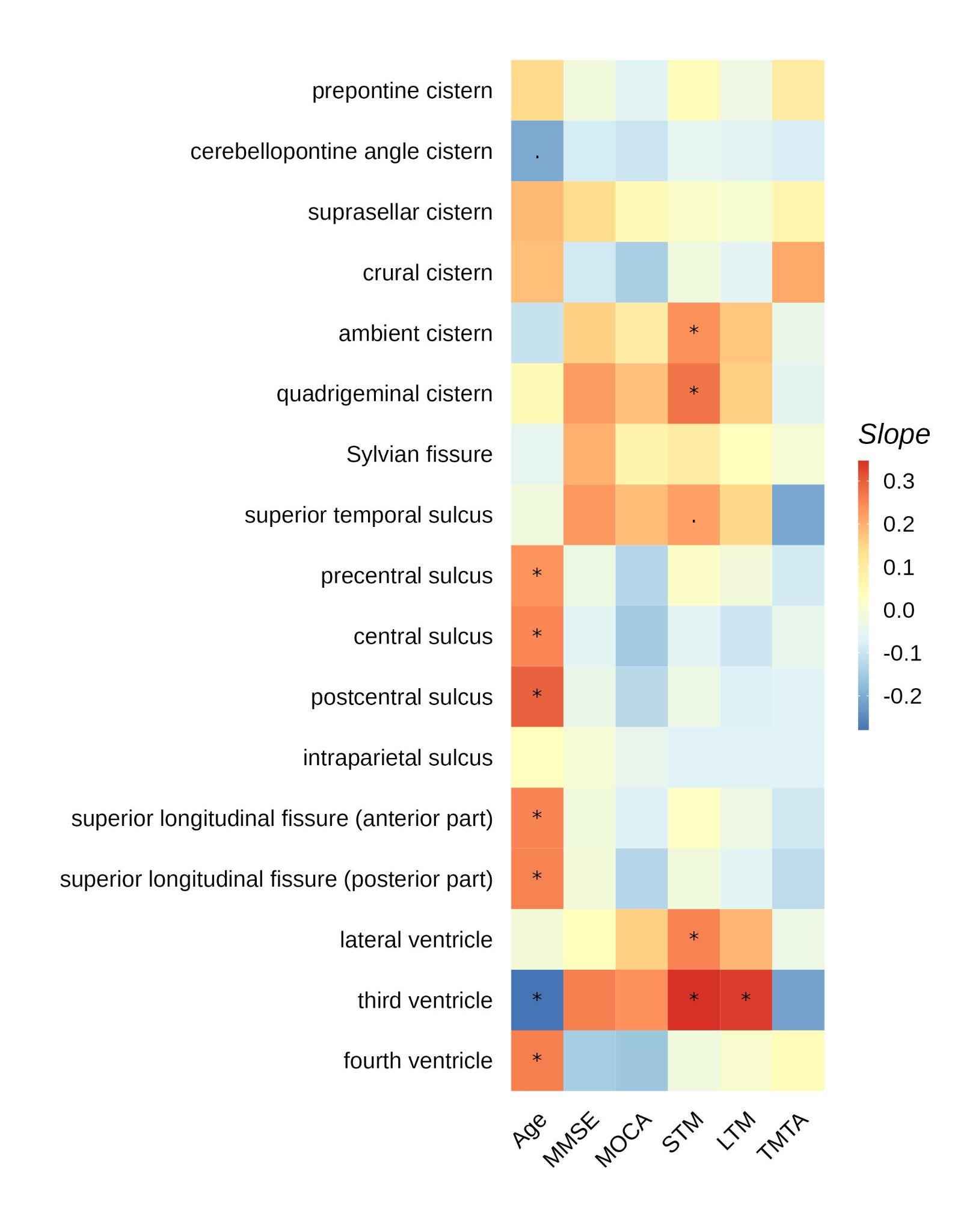


**Figure S12**. Linear regression slopes between MPD in different CSF regions with cognitive performance corrected for age, sex, years of education and intraparenchymal volume. P values were labelled as: ***: <0.001, **: 0.001-0.01, *: 0.01-0.05, .: 0.05-0.1. Higher scores in MMSE, MOCA, STM and LTM indicate better cognitive performance. Shorter TMTA time indicates better processing speed.


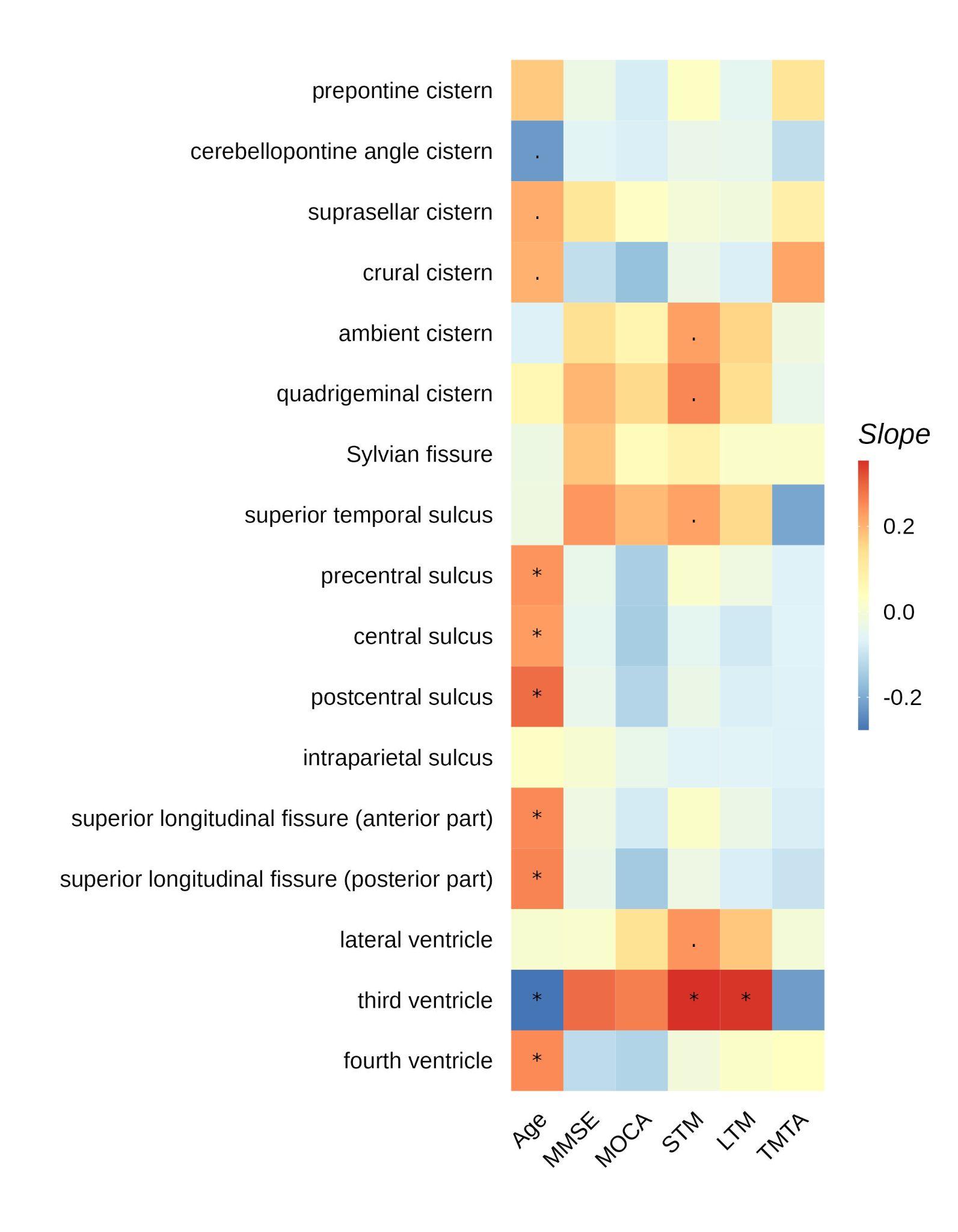


**Figure S13**. Linear regression slopes between MPD in different CSF regions with cognitive performance corrected for age, sex, years of education and intraparenchymal volume fraction. P values were labelled as: ***: <0.001, **: 0.001-0.01, *: 0.01-0.05, .: 0.05-0.1. Higher scores in MMSE, MOCA, STM and LTM indicate better cognitive performance. Shorter TMTA time indicates better processing speed.


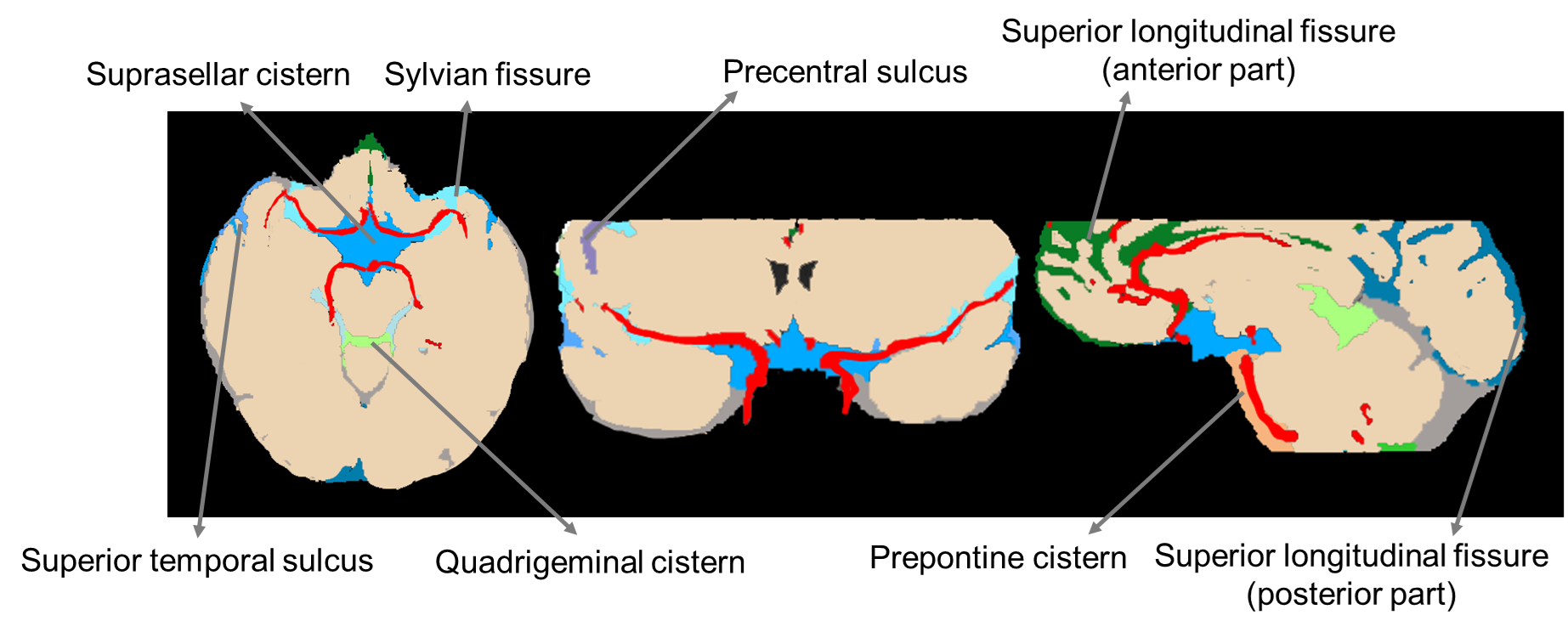


**Figure S14**. Location of sulci and cisterns in relation to major cerebral arteries (in red).
